# Indocyanine Green (ICG) Fluorescence vs. Tc-99m Lymphoscintigraphy: Optimizing Sentinel Lymph Node Detection in Cutaneous Melanoma—A Systematic Review and Meta-Analysis

**DOI:** 10.3390/jcm15031145

**Published:** 2026-02-02

**Authors:** Matteo Matteucci, Antonio Pesce, Bruno Cirillo, Lorenza Zampino, Riccardo Masserano, Salvatore Guarino, Luca Properzi, Vito D’Andrea, Roberto Cirocchi

**Affiliations:** 1 Department of Medicine and General Surgery, University of Milan, 20122 Milan, Italy; 2 Department of Surgery, Azienda Unità Sanitaria Locale (AUSL) Ferrara, 44121 Ferrara, Italy; antonio.pesce@unife.it; 3 Department of Surgery, Sapienza University, 00161 Rome, Italyvito.dandrea@uniroma1.it (V.D.); 4 Department of Surgery, Ospedale G. Fornaroli, ASST Ovest Milanese, 20013 Magenta, Italy; 5 Department of Surgery, Ospedale San Paolo, ASST Santi Paolo e Carlo, 20142 Milan, Italy; 6 Department of General Surgery, IRCCS Multimedica Sesto San Giovanni, 20099 Sesto San Giovanni, Italy; 7 Department of Medicine and Surgery, University of Perugia, 06129 Perugia, Italy

**Keywords:** melanoma, sentinel lymph node, indocyanine green, technetium-99m, lymphoscintigraphy, meta-analysis, fluorescence imaging

## Abstract

**Background**: Sentinel lymph node (SLN) biopsy has emerged as a cornerstone in melanoma staging, offering targeted evaluation of regional lymphatic spread and guiding therapeutic decision-making. Traditionally, SLN mapping relies on lymphoscintigraphy using technetium-99m (Tc-99m) radiocolloid, but in recent years, indocyanine green (ICG) fluorescence imaging has emerged as a promising alternative. The aim of this review is to evaluate the diagnostic accuracy of ICG–near-infrared (NIR) imaging compared to standard Tc-99m lymphoscintigraphy in SLN biopsy (SLNB). **Methods**: A systematic review and meta-analysis were conducted, including 12 studies. The primary outcome was the false-negative rate; secondary outcomes included the total number of sentinel lymph nodes (SLNs) identified by ICG–NIR imaging and Tc-99m lymphoscintigraphy, the number of metastatic SLNs detected by each method, and the number of patients with metastatic disease. The statistical analysis for dichotomous variables was performed using the “Odds Ratio” (O.R.) calculated with the Mantel–Haenszel method. For continuous variables, the analysis utilized the “Mean Difference” calculated by the inverse variance method. All data are presented with a 95% confidence interval (CI). **Results**: ICG was associated with a significantly higher number of SLNs identified compared to Tc-99m (O.R.: 0.41, 95% CI: 0.34–0.49; *p* < 0.00001), while no significant differences were found in the detection of metastatic nodes, either as a proportion of total SLNs (O.R.: 1.04, 95% CI: 0.86–1.25; *p* = 0.68) or relative to total positive nodes (O.R.: 0.36, 95% CI: 0.16–0.81; *p* = 0.01). No statistically significant differences between the two techniques were found in the detection of metastatic patients (OR: 0.80, 95% CI: 0.31–2.03, *p* = 0.33) and in the total number of false-negative patients missed (risk difference (RD): 0.03, 95% CI: −0.04 to 0.09, *p* = 0.93). **Conclusions**: While ICG identifies a higher number of SLNs compared to Tc-99m, its ability to detect metastatic involvement is comparable between the two modalities. No significant differences were observed in the proportion of metastatic SLNs, the total number of positive nodes detected, the number of metastatic patients identified, and the false-negative rate. Given its favorable profile, ICG could represent a reliable alternative or adjunct to Tc-99 in SLNB. However, prospective studies are warranted to validate its standalone diagnostic role.

## 1. Introduction

According to data from the Global Cancer Observatory (GLOBOCAN) [1], melanoma accounts for more than 300,000 new cases and over 50,000 deaths worldwide each year, with the highest incidence rates observed in Europe, North America, and Oceania. Despite advances in early detection and treatment, the global incidence of melanoma continues to rise, underscoring the need for accurate staging strategies and optimized management pathways. It is responsible for the majority of skin cancer-related mortality due to its strong propensity for regional and distant metastasis. In patients with clinically localized disease, regional lymph node involvement remains one of the most powerful prognostic factors influencing recurrence risk, disease-specific survival, and treatment decisions. Consequently, precise assessment of lymphatic spread is essential, particularly in early-stage melanoma [1,2].

Sentinel lymph node biopsy (SLNB) has emerged as a cornerstone in staging early-stage melanoma, offering targeted evaluation of regional lymphatic spread and guiding therapeutic decision-making [3]. Traditionally, sentinel lymph node (SLN) mapping relies on lymphoscintigraphy using technetium-99m (Tc-99m) radiocolloid, often combined with vital blue dye.

However, Tc-99m-based lymphoscintigraphy presents several inherent limitations. The technique requires access to nuclear medicine facilities, specialized personnel, and strict regulatory compliance, which may limit its availability in some centers. Additionally, preoperative imaging provides limited spatial resolution and lacks real-time intraoperative feedback, potentially complicating SLN localization, particularly in anatomically complex regions such as the head and neck. Patient exposure to ionizing radiation, although low, remains a consideration.

In recent years, indocyanine green (ICG), a fluorescent dye visualized with near-infrared (NIR) imaging, has gained attention as an alternative or adjunct to standard techniques. ICG offers several practical and safety advantages, including higher spatial resolution during surgery, lack of ionizing radiation, and simplified operative logistics [4,5,6,7]. However, evidence regarding its diagnostic accuracy for SLN identification in cutaneous melanoma remains heterogeneous, with variable study designs and outcome measures [8,9,10].

Considering these uncertainties, the present systematic review and meta-analysis were conducted to comparatively assess the diagnostic accuracy of ICG–NIR imaging and conventional Tc-99m lymphoscintigraphy for SLNB in patients with cutaneous melanoma. Specifically, this study aims to evaluate the performance of both techniques in the identification of sentinel lymph nodes (SLNs), metastatic SLNs, and patients with nodal metastases, with particular emphasis on the false-negative rate (FNR).

## 2. Materials and Methods

This systematic review and meta-analysis were conducted according to the Preferred Reporting Items for Systematic Reviews and Meta-Analysis (PRISMA) checklist (Supplementary Material) [11] and the Meta-Analysis of Observational Studies in Epidemiology (MOOSE) checklist [12] (SDC1). No ethical approval was required. The study protocol was prospectively registered in the International Prospective Register of Systematic Reviews (PROSPERO).

### 2.1. Search Strategy and Selection Criteria

A comprehensive literature search was performed across PubMed, Cochrane Library, and Google Scholar databases to identify relevant studies by two authors (M.M. and R.C.) from December 2024. The final literature search was completed in June 2025.

Keywords used for identifying the study population were based on the PICO model [13]. The search terms used to describe the study population included melanoma OR cutaneous melanoma AND sentinel lymph node biopsy OR SLNB AND sentinel lymph node OR SLN OR sentinel AND indocyanine green OR near-infrared fluorescence OR IGC OR near infrared fluorescence. Additional strategies included reviewing the references of all included articles for further relevant material.

Studies were included if they met the following criteria: (1) adult patients with a histologically confirmed diagnosis of cutaneous melanoma, (2) SLNB performed using ICG-NIR imaging and Tc-99m lymphoscintigraphy, (3) comparative data allowing evaluation of diagnostic performance, and (4) reporting at least one outcome of interest.

The exclusion criteria were studies in a language other than English, missing full-text publication, case reports, reviews, abstracts from scientific meetings, letters, animal studies, and studies regarding the use of ICG for malignancies other than cutaneous melanoma and with concomitant use of blue dyes. Any disagreements on the eligibility of full-text articles were resolved by consensus.

### 2.2. Data Extraction

All included studies were reviewed by two independent reviewers (M.M. and R.C.). Extracted data encompassed the first author, years of publication, study design, prospective or retrospective nature of the study, number of patients, mean age in years, primary tumor location, excision of SLNs based on each method, and number of metastatic SLNs identified by both methods, by ICG only and by Tc-99m only.

### 2.3. Study Outcomes

The primary outcome of interest was the false-negative rate (FNR) of ICG–NIR imaging and Tc-99m lymphoscintigraphy, respectively. A false-negative event was defined as either the occurrence of a nodal recurrence in a previously sampled sentinel lymph node (SLN) basin that was initially negative during follow-up or the detection of a metastatic SLN exclusively by the alternative modality. The FNR for ICG–NIR imaging or Tc-99m lymphoscintigraphy was calculated as follows: (number of patients with recurrence in a previously sampled negative SLN basin plus the number of patients with a metastatic SLN identified only by the alternative method) divided by the sum of these patients and the number of true-positive patients identified by the respective modality.

Secondary outcomes included the total number of SLNs identified by ICG–NIR imaging and Tc-99m lymphoscintigraphy, the number of metastatic SLNs detected by each method, and the number of patients with metastatic disease.

### 2.4. Statistical Analysis

Information on continuous variables was presented as means with standard deviations; when articles did not report these variables in this format, they were converted using the methods described in the Cochrane Handbook for Systematic Reviews of Interventions. The statistical analysis for dichotomous variables was performed using the “Odds Ratio” (O.R.) calculated with the Mantel–Haenszel method, and the results were presented as an odds ratio with a corresponding 95% confidence interval. The random-effect models were applied to all outcomes. Homogeneity among studies was evaluated by calculating the χ^2^ tests, I^2^, and *p*-values. A *p*-value greater than 0.1 and I2 values less than 50% indicated an absence of heterogeneity. I2 values between 50% and 75% suggested moderate heterogeneity, while values greater than 75% indicated high heterogeneity. Statistical analysis was carried out using Review Manager (RevMan) version 5.4.

## 3. Results

### 3.1. Literature Search

**Figure 1:**
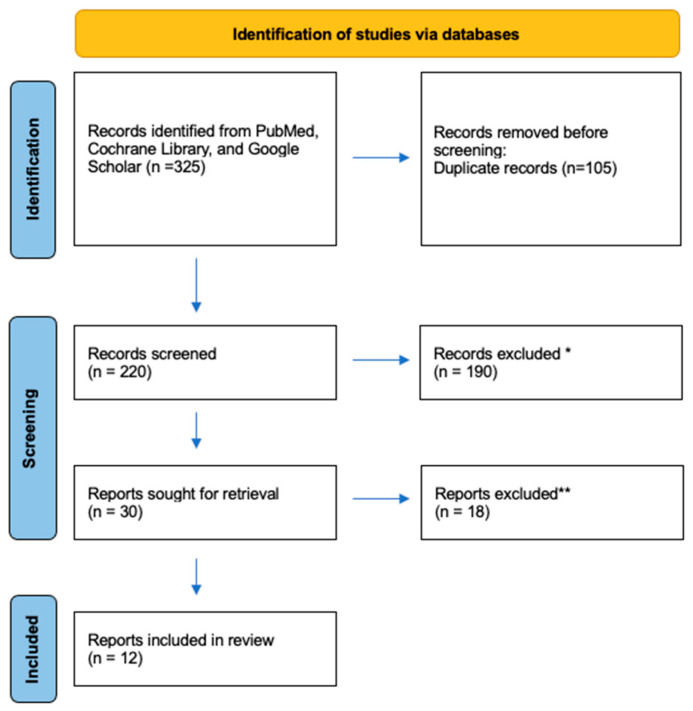
PRISMA 2020 flow diagram of the study selection process. * Records excluded for not meeting inclusion criteria; ** records excluded due to language restrictions, unavailable full text, non-original articles, animal studies, non-melanoma indications, or concomitant use of blue dyes.

A total of 325 publications were initially identified, and 105 duplicate records were removed before the primary review. The remaining 220 records were subjected to title and abstract screening by two reviewers (M.M. and R.C.) independently. Of these, 190 were excluded, and 30 full-text reports were successfully retrieved. During the secondary review, 18 reports were excluded due to the additional intraoperative use of blue dye and missing data. Thus, according to PRISMA 2020 guidelines [11], a total of 12 studies were included (Figure 1).

### 3.2. Characteristics of Included Studies

Twelve studies were included in our systematic review [14,15,16,17,18,19,20,21,22,23,24,25,26]. Characteristics of these studies are reported in Table 1. Eight of the studies included had a prospective design, while the other five represented retrospective analysis. All studies were conducted in monocentric centers between 2012 and 2025. The mean age is 60 years. Our review included a total of 1142 patients with melanoma, and there were 268 patients with primary cutaneous melanoma located in the head and neck region; in 330 cases, the primary tumor was on the trunk, while in the remaining cases, it was located on the extremities.

### 3.3. Quality Assessment of the Included Studies

**Figure 2:**
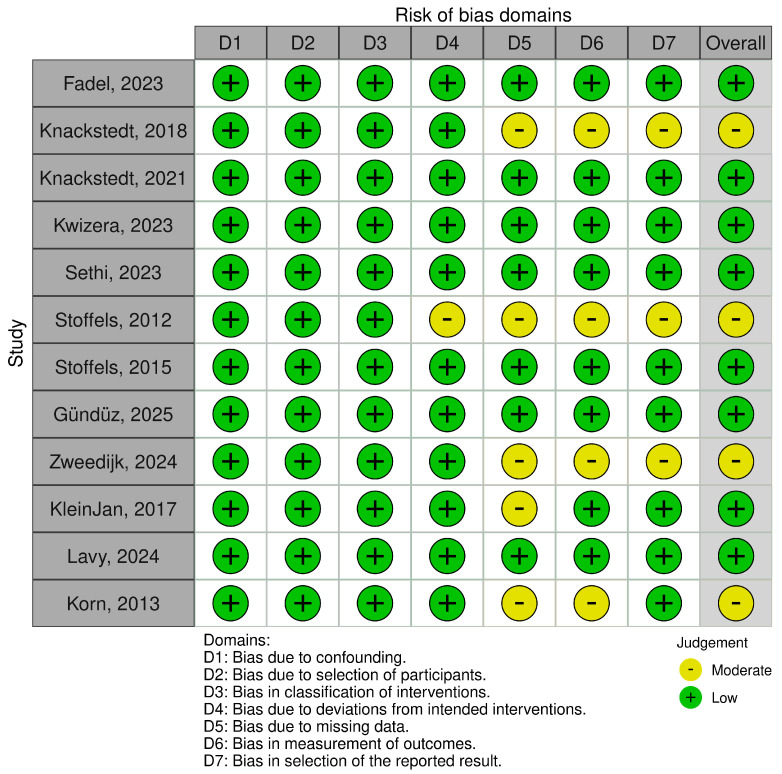
Risk of bias domains for included study CCTs using ROBINS I [14,15,16,17,18,19,20,21,22,23,24,25].

**Figure 3:**
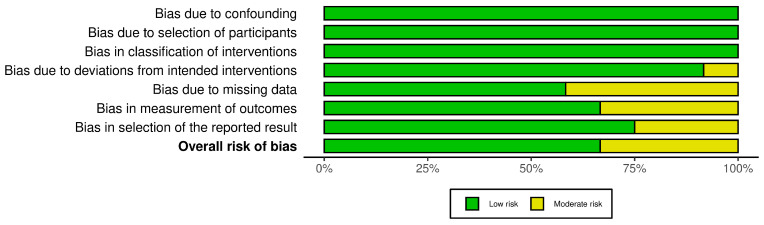
Risk of bias domains for included study CCTs using ROBINSON I.

For each included study, two reviewers (M.M. and R.C.) independently assessed the risk of bias. Disagreements were resolved by consensus. The ROBINS-I tool was used to evaluate the potential risk of bias across seven domains: bias due to confounding, bias in the selection of participants into the study, bias in the classification of interventions, bias due to deviations from intended interventions, bias due to missing data, bias in the measurement of outcomes, and bias in the selection of reported results. Only five studies presented a moderate risk of bias in domain five, while the risk of bias was moderate in four studies for domain six and three studies for domain seven. The risk of bias graph and summary are shown in Figure 2 and Figure 3, respectively.

### 3.4. Characteristics of SLNs per Tracing Method

#### 3.4.1. Total Number of SLNs Identified by Tc-99m or ICG During SLNbs

**Figure 4:**
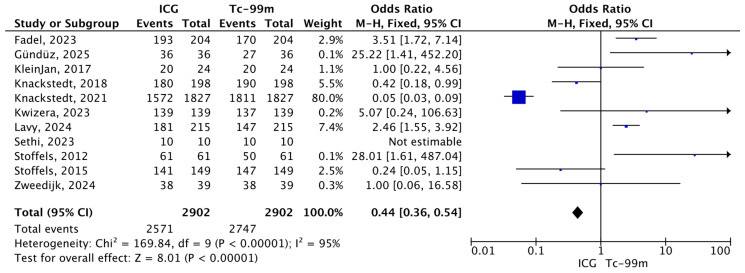
Forest plot of effect estimates for the total number of SLNs identified by Tc-99 or ICG during SLNB among all SLNs sampled. Blue square: point estimate of the effect for a single study sized according to study weight; black line: confidence interval; diamond: pooled effect estimate [14,15,16,17,18,19,20,21,22,23,24].

A total of 2902 SLNs were sampled, out of which 2571 were identified using ICG and 2747 using Tc-99m. All twelve studies reported data on sentinel lymph node identification using both tracers and were included in the meta-analysis. Pooled analysis using a fixed-effects Mantel–Haenszel model showed a significantly lower odds of non-identification with ICG–NIR imaging compared to Tc-99m (O.R.: 0.44, 95% CI: 0.36–0.54; *p* < 0.00001). However, substantial heterogeneity was observed among the included studies (I^2^ = 95%, *p* < 0.00001), indicating considerable variability in effect sizes across studies (Figure 4).

#### 3.4.2. Total Number of Metastatic SLNs Identified by Tc-99 or ICG During SLNB Among the Total Number of SLNs Excised

**Figure 5:**
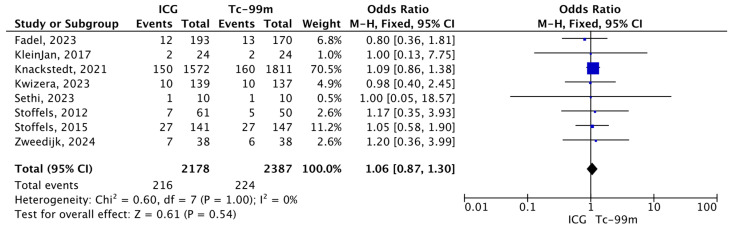
Forest plot of effect estimates for the total number of metastatic SLNs identified by Tc-99 or ICG during SLNB among all SLNs sampled. Blue square: point estimate of the effect for a single study sized according to study weight; black line: confidence interval; diamond: pooled effect estimate [14,16,17,18,19,20,23,24].

A total of 2178 and 2387 sentinel lymph nodes were identified using indocyanine green and technetium-99, respectively. Eight studies reported data on sentinel lymph node identification using both tracers and were consequently included in the meta-analysis. The forest plot evaluates the proportion of positive SLNs among the total number of SLNs excised, comparing ICG-NIR and Tc-99 in patients with cutaneous melanoma. The overall estimate suggests no statistically significant difference between the two techniques in terms of the positivity rate. Confidence intervals for most individual studies cross the line of no effect, and the pooled result supports equivalence between the two methods in detecting metastatic involvement of SLNs (O.R.: 1.06, 95% CI: 0.87–1.30; *p* = 0.54) (Figure 5).

#### 3.4.3. Total Number of Metastatic SLNs Identified by Tc-99 or ICG During SLNB Among the Total Number of Positive SLNs Excised

**Figure 6:**
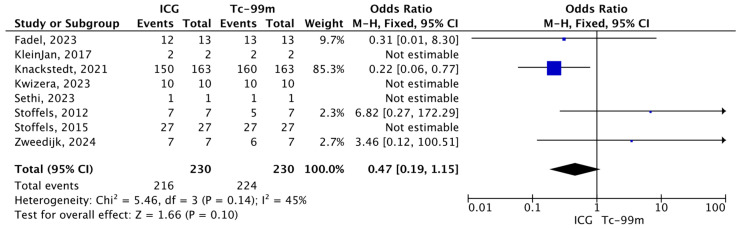
Forest plot of effect estimates for the total number of metastatic SLNs identified by Tc-99 or ICG relative to the total number of metastatic SLNs. Blue square: point estimate of the effect for a single study sized according to study weight; black line: confidence interval; diamond: pooled effect estimate [14,16,17,18,19,20,23,24].

The forest plot analyzes the proportion of metastatic SLNs identified by each technique, indocyanine green (ICG) and technetium-99m (Tc-99), relative to the total number of positive SLNs detected. Overall, 210 positive lymph nodes were identified using ICG and 224 using Tc-99m. The overall pooled estimate demonstrates no statistically significant difference between ICG and Tc-99 in their ability to identify metastatic SLNs (O.R.: 0.47, 95% CI: 0.19–1.15; *p* = 0.14) (Figure 6).

#### 3.4.4. Total Number of Metastatic Patients Identified Using ICG and Tc-99m Out of the Total Number of Metastatic Patients

**Figure 7:**
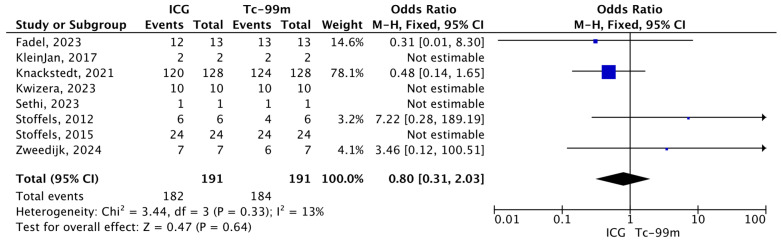
Forest plot of effect estimates for the total number of metastatic patients identified by Tc-99 or ICG relative to the total number of metastatic patients. Blue square: point estimate of the effect for a single study sized according to study weight; black line: confidence interval; diamond: pooled effect estimate [14,16,17,18,19,20,23,24].

The pooled analysis assessed the proportion of metastatic patients identified by indocyanine green compared with technetium-99m (Tc-99m) among the total number of patients with nodal metastases. Overall, 182 metastatic patients were identified using ICG and 191 using Tc-99m. The meta-analysis did not demonstrate a statistically significant difference between the two techniques (O.R.: 0.80, 95% CI: 0.31–2.03, *p* = 0.33). Low heterogeneity was observed across studies (I^2^ = 13%; χ^2^ = 3.44, *p* = 0.33), indicating good consistency among the included studies (Figure 7).

#### 3.4.5. Total Number of False-Negative Patients Missed by ICG and Tc-99m Out of the Total Number of True-Positive and False-Negative Patients

**Figure 8:**
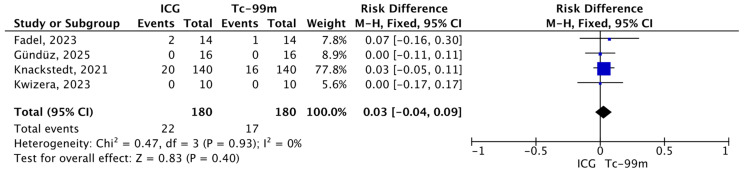
Forest plot of effect estimates for the total number of false-negative patients missed by Tc-99 or ICG relative to the total number of false-negative and true-positive patients. Blue square: point estimate of the effect for a single study sized according to study weight; black line: confidence interval; diamond: overall effect estimate [14,16,17,21].

The pooled analysis evaluated the proportion of false-negative patients missed by indocyanine green (ICG) compared with technetium-99m (Tc-99m) among the total number of false-negative and true-positive cases. Overall, 22 events were observed in the ICG group and 18 in the Tc-99m group, out of 180 patients per group. The meta-analysis demonstrated no statistically significant difference between the two techniques (R.D.: 0.03, 95% CI: −0.04–0.09, *p* = 0.93). No heterogeneity was detected across studies (I^2^ = 0%; χ^2^ = 0.47, *p* = 0.93) (Figure 8).

## 4. Discussion

SLNB remains a pivotal component in the staging and prognostic stratification of patients with cutaneous melanoma. The status of the sentinel lymph node (SLN) represents the most powerful predictor of disease-free and melanoma-specific survival. Multiple studies, including the Multicenter Selective Lymphadenectomy Trial I (MSLT-I) [27], have consistently demonstrated that SLN status is the most powerful predictor of disease-free and melanoma-specific survival: 5-year survival in SLN-negative patients was around 90% versus approximately 72% in SLN-positive patients (*p* < 0.001). Consequently, the accuracy and reliability of SLN mapping techniques are of paramount clinical importance. In recent years, indocyanine green (ICG) fluorescence imaging has gained increasing attention as an alternative or adjunct to conventional technetium-99m (Tc-99m)-based lymphoscintigraphy. ICG can offer several practical advantages. These include the absence of ionizing radiation, simplified logistics without the need for nuclear medicine facilities, real-time visualization of lymphatic flow, and the availability of portable imaging systems. Such features may be particularly advantageous in centers without access to Tc-99m, in resource-limited settings, or in specific anatomical regions, such as the head and neck, where complex lymphatic drainage patterns can complicate SLN identification. On the other hand, its role remains a matter of ongoing debate in the current literature. One major point of controversy relates to the limited tissue penetration of near-infrared fluorescence, which may reduce the sensitivity of ICG for identifying deeply located lymph nodes or for preoperative transcutaneous mapping, particularly in patients with higher body mass index [28]. These factors may be due to the limited transcutaneous visibility of ICG, which is well documented in the literature. In this context, it is crucial to distinguish between transcutaneous detection, which refers to the ability to localize the sentinel lymph node through intact skin prior to incision, and intraoperative detection, which occurs after surgical exposure. While transcutaneous detection with ICG is highly variable and often inferior to Tc-99m, intraoperative detection rates are consistently high once the lymphatic basin is exposed, allowing direct visualization of fluorescent lymphatic channels and nodes.

In fact, the penetration depth of near-infrared (NIR) fluorescence is limited, generally ranging between 1.0 [29] and 1.5 cm [20] due to absorption and scattering in biological tissues, which can compromise the ability to localize SLN before skin incision, particularly in patients with higher body mass index or deeply situated nodes. This limited tissue penetration has been identified as a factor contributing to lower transcutaneous detection rates of ICG in cutaneous melanoma, with studies reporting detection rates as low as 21.9% when assessed prior to incision, thereby questioning its feasibility as a sole preoperative mapping tool for SLN localization in its current form [30]. Several investigations have contrasted these results, showing that ICG identified the SLN region transcutaneously with higher sensitivity in selected cohorts, most notably the prospective diagnostic sensitivity study by Lese et al. In this cohort, transcutaneous detection of SLN with ICG before skin incision was achieved in 96.1% of cases for SLN location and in 79.4% for the number of SLNs identified [28].

The present meta-analysis, including 12 studies, provides an updated and comprehensive synthesis of the available evidence comparing these two techniques. Our results demonstrate that ICG is associated with a higher detection rate of sentinel lymph nodes compared to Tc-99m-based lymphoscintigraphy. However, an important finding of the present meta-analysis is the extremely high heterogeneity observed in the pooled analysis of total SLN identification (I^2^ = 95%). Such heterogeneity raises concerns regarding the robustness and clinical generalizability of the pooled estimate favoring ICG. Several factors are likely contributors, including substantial variations in ICG protocols (dose, injection volume, timing of administration, and imaging systems), heterogeneity in patient populations, and differences in anatomical tumor sites, particularly between head and neck melanoma and trunk or extremity lesions. These sources of variability limit the ability to draw definitive conclusions regarding the superiority of ICG in SLN detection. Therefore, although ICG appears to identify a higher number of sentinel lymph nodes, this finding should be interpreted with caution, considering the marked heterogeneity across studies.

Importantly, despite the higher number of SLNs retrieved with ICG, no clinically meaningful differences were observed between ICG and Tc-99m in terms of metastatic SLN detection. Specifically, the proportion of metastatic SLNs among all excised nodes and the proportion of positive nodes detected by each technique relative to the total number of metastatic SLNs were comparable. Furthermore, our analysis demonstrated no significant difference in the false-negative rate between ICG–NIR imaging and Tc-99m lymphoscintigraphy. This finding suggests that both techniques provide comparable diagnostic accuracy in correctly classifying patients as metastatic or non-metastatic. However, these findings should be interpreted with caution. In fact, recurrence represents a time-dependent oncologic outcome influenced by follow-up duration, surveillance intensity, and tumor biology. Importantly, follow-up duration was not standardized across studies and was often shorter or incompletely reported in retrospective cohorts, increasing the risk of underestimating late nodal recurrences. Consequently, FNR estimates may underestimate the true false-negative rate, particularly for studies with limited long-term oncologic follow-up. These considerations further support the need for prospective studies with standardized follow-up and uniform FNR definitions to accurately assess the long-term oncologic reliability of ICG-based SLN mapping.

Furthermore, only three of the included studies explicitly reported a distinction between in vivo intraoperative detection and ex vivo assessment of sentinel lymph nodes. In most studies, sentinel lymph nodes were classified as ICG- or Tc-99m-positive based on ex vivo evaluation on the side table, without clearly documenting whether the nodes were identifiable intraoperatively by each modality alone. In fact, ex vivo fluorescence confirmation does not necessarily reflect the ability of ICG to reliably guide intraoperative SLN localization as a standalone modality. Moreover, in many studies, ICG was used in combination with Tc-99m, which further limits the possibility of drawing definitive conclusions regarding the independent performance of ICG.

Our results are consistent with, and expand upon, those of previously published systematic reviews and meta-analyses. In fact, according to a recent systematic review and meta-analysis [8,10], ICG-NIR appears to be a non-inferior alternative in terms of SLN detection, metastatic SLN detection, and false-negative rates. Multiple cohort studies [14,22] have confirmed that ICG-NIR, when used either with Tc-99 or blue dye, achieves detection rates comparable to or exceeding those of traditional tracers. For instance, Stoffels et al. [20] retrospectively compared traditional Tc-99m + ICG versus traditional Tc-99m + blue dye and found similar SLN detection rates (98.8% vs. 100%), with fewer nodes excised and comparable positivity rates. Similarly, Korn et al. [25] reported that ICG + Tc-99m achieved localization rates (98%) matching those of Tc-99m alone and outperforming blue dye alone (79%).

Nevertheless, several limitations must be acknowledged. Most of the included studies were monocentric and non-randomized, with a combination of prospective and retrospective designs, which may introduce selection bias and limit the generalizability of the findings. In addition, there was substantial heterogeneity in ICG protocols across studies, including differences in dye concentration, injection volume, timing of administration, imaging systems, and surgeon experience. This lack of standardization may have influenced SLN detection rates and complicates direct comparison of results across studies.

An important methodological limitation relates to how SLN detection was assessed. Only a small proportion of the included studies explicitly distinguished between true in vivo intraoperative detection and ex vivo assessment of excised lymph nodes. In most studies, SLNs were classified as ICG-positive based on ex vivo fluorescence on the side table, which does not necessarily reflect whether the node would have been identifiable intraoperatively by ICG alone. This issue is particularly relevant in studies using ICG concomitantly with Tc-99m and may lead to an overestimation of the standalone performance of ICG.

Furthermore, long-term oncologic outcomes such as recurrence rates and survival were not consistently reported, precluding definitive conclusions regarding oncologic equivalence. Finally, a notable limitation is the substantial influence of a single study, Knackstedt et al. [16], which contributed over 60% of the weight in several pooled analyses. A formal sensitivity analysis excluding this study was not performed, as its removal would have substantially reduced the available sample size and, for several outcomes, precluded meaningful quantitative synthesis. Moreover, visual inspection of forest plots did not suggest effect reversal or inconsistency driven by this study, and random-effects models were applied to mitigate the influence of large studies. However, the heavy weighting of a single study limits the robustness and generalizability of pooled estimates and should be considered when interpreting the results.

Taken together, these limitations suggest that, although ICG is a reliable and effective tool for intraoperative SLN identification, its performance as a standalone modality remains uncertain. Limitations in transcutaneous detection, the frequent reliance on ex vivo confirmation, heterogeneity in study protocols, and the lack of long-term oncologic outcomes data indicate that ICG cannot yet be considered a complete replacement for Tc-99m in all clinical scenarios. Its role is, therefore, best defined as a complementary or adjunctive technique, particularly for intraoperative guidance, while further standardized and methodologically rigorous studies are needed to clarify its potential as a primary mapping modality.

## 5. Conclusions

This meta-analysis confirms that ICG fluorescence imaging is a reliable and effective technique for intraoperative SLN visualization in cutaneous melanoma. However, the available evidence primarily supports its role as an adjunctive intraoperative tool rather than as a complete replacement for Tc-99m. The frequent reliance on ex vivo fluorescence assessment and the widespread combined use of ICG with Tc-99m limit conclusions regarding the standalone performance of ICG-NIR across all clinical scenarios.

Accordingly, conclusions suggesting equivalence between ICG-NIR and Tc-99m should be interpreted with caution, particularly in settings where preoperative or transcutaneous SLN localization is required. Further prospective studies specifically designed to evaluate ICG as a standalone modality, with standardized protocols and long-term oncologic follow-up, are needed before its routine use as a full alternative to Tc-99m can be recommended.

## Figures and Tables

**Table 1:** Characteristics of studies included.

Authors	Design	Location	N° of Patients	Mean Age (Years)	Primary Tumor Location
Kwizera 2023 [17]	Retrospective	Monocentric	52	63	Head and Neck: 11 Trunk: 20 Extremities: 21
Fadel 2023 [14]	Retrospective	Monocentric	122	60.5	Head and Neck: 13 Trunk: 38 Extremities: 71
Sethi 2023 [18]	Retrospective	Monocentric	10	65	Head and Neck: 10 Trunk: 0 Extremities: 0
Stoffels 2015 [20]	Prospective	Monocentric	80	55.5	Head and Neck: 0 Trunk: 40 Extremities: 40
Stoffels 2012 [19]	Retrospective	Monocentric	22	51.6	Head and Neck: 2 Trunk: 9 Extremities: 11
Knackstedt 2021 [16]	Prospective	Monocentric	594	61.2	Head and Neck: 136 Trunk: 163 Extremities: 295
Zweedijk 2024 [23]	Prospective	Monocentric	32	57	N.R.
KleinJan 2017 [24]	Prospective	Monocentric	8	60, 5	Head and Neck: 8 Trunk: 0 Extremities: 0
Lavy 2024 [22]	Retrospective	Monocentric	84	63	Head and Neck: 10 Trunk: 39 Extremities: 35
Korn 2013 [25]	Prospective	Monocentric	51	57.8	Head and Neck: 15 Trunk: 16 Extremities: 20
Gündüz 2025 [21]	Prospective	Monocentric	20	60	Head and Neck: 2 Trunk: 0 Extremities: 13
Knackstedt 2018 [15]	Prospective	Monocentric	61	64.3	Head and Neck: 61 Trunk: 0 Extremities: 0

## Data Availability

The data used to support the findings of this study are included within the article. The data presented in this study are available upon request.

## Supplementary Materials

The following supporting information can be downloaded at https://www.mdpi.com/article/10.3390/jcm15031145/s1, SDC1: PRISMA Checklist 2020.
